# A secondary head-to-head comparison of low-intensity focused ultrasound and repetitive transcranial magnetic stimulation for motor recovery after stroke

**DOI:** 10.1371/journal.pone.0348030

**Published:** 2026-04-24

**Authors:** Shuhong Zheng, Renxiu Bian, Haixin Song, Zhiping Liao, Ting Gao, Min Yan, Heqing Huang, Zuodong Lou, Fangchao Wu, Jianhua Li

**Affiliations:** 1 Hangzhou Women’s Hospital (Hangzhou Maternity and Child Health Care Hospital), Hangzhou, China; 2 Department of Physical Medicine and Rehabilitation, Sir Run Run Shaw Hospital, Zhejiang University School of Medicine, Hangzhou, China; 3 Department of Physical Medicine and Rehabilitation, Shaoxing Shangyu Hospital of Traditional Chinese Medicine, Shaoxing, China; Hangzhou Normal University, CHINA

## Abstract

**Background:**

Low-intensity focused ultrasound (LIFU) is a non-invasive neuromodulation technique with high spatial precision and the ability to reach deeper brain regions, offering potential advantages for post-stroke rehabilitation. Repetitive transcranial magnetic stimulation (rTMS) is a widely adopted non-invasive brain stimulation technique that modulates cortical excitability to promote neuroplasticity. However, direct head-to-head comparisons between these two modalities for post-stroke motor recovery remain limited.

**Objective:**

To perform a secondary head-to-head comparison of LIFU and repetitive transcranial magnetic stimulation (rTMS) for motor recovery after stroke, based on a prospectively registered randomized controlled trial.

**Methods:**

This secondary analysis included patients with subacute stroke who received two weeks of standard rehabilitation combined with either LIFU (n = 25) or rTMS (n = 25) targeting the ipsilesional primary motor cortex. LIFU parameters: 0.5 MHz, spatial-peak pulse-average intensity (ISPPA) 10.2 W/cm² (free-field), pulse duration 0.2 ms, duty cycle 20%, 20 minutes per session, five days per week for two weeks (10 sessions total). rTMS parameters: 10 Hz, 80% resting motor threshold, 1,000 pulses per session (20 trains of 5 seconds), 20 minutes per session, five days per week for two weeks (10 sessions total). Motor outcomes were assessed using the Fugl–Meyer Assessment (FMA; upper and lower extremities), Modified Barthel Index (MBI), and Brunnstrom stages. Resting-state functional near-infrared spectroscopy (fNIRS) was used to evaluate cortical activity and functional connectivity before and after the intervention. Primary analyses were conducted in the intention-to-treat (ITT) population (n = 50), with completer analyses (n = 43) performed as sensitivity analyses.

**Results:**

Both groups showed significant within-group improvements in FMA and MBI after the intervention (all p < 0.001), and changes in Brunnstrom stages were also uniformly significant. No statistically significant between-group differences were observed in post-intervention FMA, MBI, or Brunnstrom stages (all p > 0.05), and completer analyses yielded consistent between-group conclusions. In contrast, change-from-baseline analyses demonstrated greater improvements in FMA scores in the LIFU group compared with the rTMS group (ΔFMA upper limb: median 7 [IQR 3–10.5] vs. 2 [1–3], p = 0.001; lower limb: 3 [1–4.5] vs. 1 [0–1.5], p < 0.001). Exploratory fNIRS analyses revealed modality-specific patterns: prefrontal fractional amplitude of low-frequency fluctuations (fALFF) increased significantly in the LIFU group (p = 0.002) but not in the rTMS group, while functional connectivity changes did not remain significant after correction for multiple comparisons.

**Conclusion:**

LIFU and rTMS were associated with comparable short-term motor outcomes in subacute stroke. Differences observed in change-from-baseline motor improvements and exploratory neuroimaging measures suggest potential divergence in recovery dynamics and cortical modulation, warranting further investigation in larger, longitudinal studies.

**Trial registration:**

This study was derived from a prospectively registered, three-arm randomized controlled trial in the Chinese Clinical Trial Registry (ChiCTR2500114687). The present manuscript reports a secondary head-to-head comparison between the two neuromodulation intervention arms.

## 1. Introduction

Stroke remains one of the leading causes of death and disability worldwide. According to the latest Global Burden of Disease 2021 estimates, approximately 11.9 million new strokes and 7.25 million stroke-related deaths occurred in 2021 [1], underscoring the continuing and growing global health burden. Motor dysfunction is the primary consequence of a stroke. Recent studies show that approximately 80% of stroke survivors experience motor impairment during the acute phase. Moreover, over 55% to 75% of these individuals continue to face significant motor deficits in the long term, which significantly impacts their quality of life [2,3].

Functional recovery after stroke fundamentally depends on neuroplasticity and the reorganization of neural circuits, processes that involve synaptogenesis, axonal sprouting, dendritic remodeling, and large-scale network reconnection [4,5]. In recent years, non-invasive brain stimulation techniques, most notably repetitive transcranial magnetic stimulation (rTMS), have emerged as promising adjuncts to standard rehabilitation by modulating cortical excitability and promoting activity-dependent plasticity to support network reorganization [6,7]. Despite growing clinical adoption, rTMS is subject to inherent physical constraints, including limited spatial focality and restricted penetration depth, which may limit its capacity to directly modulate deeper subcortical components of motor networks implicated in post-stroke recovery [8].

Low-intensity focused ultrasound (LIFU) is an emerging non-invasive neuromodulation technique that employs non-ionizing ultrasonic waves to achieve millimeter-level spatial precision and the capacity to reach deeper brain structures. These physical characteristics have generated growing interest in LIFU as a potential approach for modulating motor-related neural circuits that may be less accessible to standard non-invasive stimulation methods [9]. As the field advances, LIFU is gradually transitioning from preclinical investigation toward early clinical application in stroke rehabilitation. A recent phase I feasibility study in stroke survivors demonstrated that single-session LIFU delivered to the ipsilesional motor cortex at intensities up to 8 W/cm² was safe and associated with exploratory improvements in motor performance and corticospinal excitability [10]. In addition, preclinical and translational studies suggest that LIFU can influence neurovascular, neuroinflammatory, and synaptic plasticity-related processes. Collectively, these findings indicate that LIFU represents a promising neuromodulation modality for further investigation in the context of post-stroke motor recovery [11–13].

Despite growing interest in non-invasive neuromodulation for post-stroke motor recovery, direct head-to-head randomized comparisons between low-intensity focused ultrasound and rTMS remain limited, particularly with respect to their differential effects on functional connectivity (FC) and large-scale neural network organization. To address this gap, the present study performs a secondary head-to-head comparison of LIFU and rTMS derived from a prospectively registered randomized controlled trial. Motor recovery is assessed using standardized clinical scales, while functional near-infrared spectroscopy (fNIRS) is employed to characterize treatment-associated changes in cortical FC and network-level patterns. By integrating clinical and neuroimaging measures, this study aims to provide comparative evidence on the neuromodulatory profiles of LIFU and rTMS in the context of post-stroke motor rehabilitation.

## 2. Materials and methods

### 2.1. Study design and participants

This study reports a secondary head-to-head analysis derived from a prospectively conducted, single-blind, three-arm randomized controlled trial carried out between October and December 2024 at the Department of Physical Medicine and Rehabilitation, Sir Run Run Shaw Hospital, Zhejiang University School of Medicine. The original trial was designed as a three-arm study (standard rehabilitation alone, standard rehabilitation + LIFU, or standard rehabilitation + rTMS; 1:1:1 allocation) with a target sample size of 75 participants (25 per group). Sample size estimation was performed using G*Power 3.1 [9], assuming a medium effect size (Cohen’s d = 0.5), a two-sided α level of 0.05, and a statistical power of 0.80. The present manuscript reports a secondary comparison between the two neuromodulation arms (LIFU and rTMS); participants allocated to the standard rehabilitation-only group were excluded from the current analysis. All data used for the present secondary analysis were accessed on December 1,2024 in fully anonymized form. The study protocol was approved by the Ethics Committee of Sir Run Run Shaw Hospital (Approval No. 2024-Yan-0511). Written informed consent was obtained from all participants or their legal representatives prior to enrolment; the ethics committee did not waive the requirement for informed consent.

For the present analysis, participants allocated to the LIFU and rTMS arms were included (n = 50; 25 per group). This head-to-head comparison represents a secondary analysis that was not independently powered. Post-hoc power analysis indicated that the current sample size provides approximately 78% power to detect a medium effect size (Cohen’s d = 0.5) for between-group comparisons, approaching but not reaching the conventional 80% threshold.

#### 2.1.1. Eligibility criteria.

Participants were eligible for inclusion if they met the following criteria: (1) first-ever stroke confirmed by cranial computed tomography (CT) or magnetic resonance imaging (MRI), with stroke onset within 6 months and documented neurological stability for at least 2 weeks prior to enrollment; (1a) For patients with hemorrhagic stroke: intracranial hemorrhage confirmed by CT/MRI with complete resolution of hematoma as evidenced by follow-up imaging, absence of acute or unstable bleeding, and no signs of increased intracranial pressure or mass effect; (1b) For patients with ischemic stroke: confirmed cerebral infarction without hemorrhagic transformation or mass effect; (2) age between 40 and 80 years; (3) unilateral limb motor dysfunction without severe aphasia; (4) preserved consciousness and no significant cognitive impairment, defined as a Mini-Mental State Examination score ≥ 23, allowing adequate cooperation during interventions and assessments; and (5) Brunnstrom stage ≥ I and a Modified Ashworth Scale grade ≤ 3. Baseline characteristics, including time since stroke onset, were comparable between groups (P > 0.05), minimizing potential confounding from disease stage heterogeneity.

#### 2.1.2. Exclusion criteria.

Participants were excluded if they had: (1) a prior history of central nervous system disorders, including previous intracerebral hemorrhage, cerebral infarction, traumatic brain injury, or transient ischemic attack; (2) bilateral hemispheric involvement following stroke; (3) severe aphasia, dysarthria, cognitive impairment, or impaired consciousness that could interfere with rehabilitation or outcome assessment; or (4) medical conditions that could pose risks during rehabilitation or neuromodulation, including severe psychiatric disorders, epilepsy, significant cardiopulmonary disease, implanted metal devices, or pregnancy; (5) Active intracranial hemorrhage, subarachnoid hemorrhage with unresolved aneurysm or vascular malformation, or any condition posing high risk of recurrent bleeding; (6) Uncontrolled hypertension (systolic blood pressure > 180 mmHg or diastolic blood pressure > 110 mmHg) or coagulopathy (INR > 1.5 or platelet count < 100,000/μL) in patients with history of hemorrhagic stroke.

#### 2.1.3. Withdrawal criteria.

Participants were withdrawn from the study if they voluntarily requested discontinuation, experienced clinical deterioration, or were lost to follow-up during the intervention period.

### 2.2. Randomization and blinding

Participants were randomly allocated in a 1:1:1 ratio to one of three intervention groups: standard rehabilitation alone, standard rehabilitation combined with LIFU, or standard rehabilitation combined with rTMS. Randomization was performed using a computer-generated random sequence by an independent researcher who was not involved in participant recruitment, intervention delivery, or outcome assessment. Group assignments were concealed in sealed, opaque envelopes and were revealed only after baseline assessments had been completed.

Owing to the nature of the neuromodulation interventions, participants and treating therapists were not blinded to group allocation. However, outcome assessors and data analysts were blinded to treatment assignment throughout the study period.

The present manuscript reports a secondary head-to-head comparison between the two neuromodulation intervention arms (LIFU and rTMS). Participants allocated to the standard rehabilitation-only group were not included in the analyses presented here.

### 2.3. Intervention protocol

All participants received standard rehabilitation for two consecutive weeks. According to group allocation, standard rehabilitation was delivered either alone or in combination with an adjunctive neuromodulation intervention. In the present analysis, participants assigned to the low-intensity focused ultrasound (LIFU) or rTMS arms were included.

For both neuromodulation groups, stimulation was applied over the ipsilesional primary motor cortex (M1). Intervention schedules were time-matched across groups: each stimulation session lasted 20 minutes and was administered five days per week for two weeks.

#### 2.3.1. Safety protocol and monitoring.

For all participants, comprehensive safety assessments were conducted prior to enrollment and throughout the intervention period. For patients with hemorrhagic stroke, pre-treatment CT or MRI confirmed complete resolution of the hematoma and absence of mass effect or edema. Vital signs (blood pressure, heart rate) were monitored before and after each neuromodulation session. Any adverse events, including headache, nausea, or neurological deterioration, were documented. rTMS was contraindicated in patients with intracranial metallic implants or uncontrolled seizure disorders. LIFU parameters were maintained within established safety limits (derated ISPPA 3.4 W/cm^2^, MI < 1.3) to minimize thermal or mechanical risks.

#### 2.3.2. Standard rehabilitation.

Standard rehabilitation consisted of individualized physical therapy (PT) and occupational therapy (OT). PT encompassed neuromuscular electrical stimulation (NMES), joint mobilization, muscle strengthening, transfer and balance training, and limb positioning. OT focused primarily on activities of daily living practice. Rehabilitation sessions were administered by licensed therapists, with each session lasting approximately 2 hours (including both PT and OT components) and delivered five days per week for two consecutive weeks. All participants received the same standardized rehabilitation protocol, regardless of neuromodulation group allocation.

#### 2.3.3. LIFU intervention.

LIFU stimulation was delivered using a single-element transcranial focused ultrasound system (Model FUT56067, Beijing Ruoao Medical Technology Co., Ltd.; fundamental frequency 0.5 MHz, focal distance 50 mm, lateral focal width 2 mm, axial focal length 23 mm). The ultrasound transducer was coupled to the scalp using ultrasound gel. The ipsilesional primary motor cortex was targeted based on individual structural T1-weighted magnetic resonance imaging and neuronavigation software [14].

Stimulation parameters were as follows: acoustic output power of 4.0 W, spatial-peak pulse-average intensity (ISPPA) of 10.2 W/cm^2^ (free-field), pulse duration of 0.2 ms, and pulse repetition period of 1 ms (pulse repetition frequency of 1 kHz; duty cycle of 20%). Sonication was delivered in 1-s trains with an inter-stimulus interval of 0.2 s. Accounting for skull attenuation, in situ acoustic parameters were estimated based on literature-reported attenuation models. The derated ISPPA and ISPTA were estimated to be 3.4 W/cm^2^ and 0.68 W/cm^2^, respectively. Peak rarefactional pressure and mechanical index were derived accordingly, and the estimated temperature rise was less than 0.5°C [15,16].

Participants remained awake during stimulation, and clinical status was continuously monitored throughout each session.

#### 2.3.4. rTMS intervention.

rTMS was delivered using a figure-of-eight coil (PF8P-100TU; Max Medical Technology Co., Ltd., Zhengzhou, China) connected to a TMS-100B stimulator. The ipsilesional primary motor cortex (M1) was localized according to established clinical procedures. Briefly, the Cz position was first identified using the international 10–20 EEG system. As an initial estimate, the coil was positioned approximately 5 cm lateral to Cz toward the hemisphere ipsilateral to the stimulated cortex, corresponding to the approximate location of the hand motor area [17,18]. This landmark-based approach was subsequently refined using single-pulse TMS to identify the precise scalp location (hot spot) eliciting the largest motor-evoked potentials (MEPs) in the contralateral first dorsal interosseous muscle. The M1 hot spot was defined as the site with the lowest motor threshold and largest MEP amplitude. If MEPs were absent, contralesional RMT was used.

For stimulation, the figure-of-eight coil was positioned with its center over the M1 hot spot, tangential to the scalp surface. The coil handle was oriented posterior-laterally at approximately 45° to the midline (perpendicular to the central sulcus), inducing a posterior-to-anterior current flow in the underlying cortex. Resting motor threshold was determined using single-pulse TMS and was defined as the lowest stimulator output capable of eliciting motor-evoked potentials ≥ 50 μV in at least 5 of 10 consecutive trials [19]. High-frequency rTMS (10 Hz) was then applied over the ipsilesional M1. Stimulation parameters were set at an intensity of 80% of the resting motor threshold, with a train duration of 5 s, an inter-train interval of 25 s, and 20 trains per session, yielding a total of 1000 pulses per session. The active stimulation period (including inter-train intervals) totaled approximately 10 minutes. Each session lasted approximately 20 minutes [20], which encompasses the active stimulation trains, inter-train intervals, and routine clinical setup procedures (including coil positioning, patient positioning, skin preparation, and M1 localization).

Participants remained awake throughout the stimulation sessions and were continuously monitored for motor responses and clinical status.

### 2.4. Outcome measures

Outcome assessments were conducted at baseline and immediately after the 2-week intervention period. The primary endpoint was motor recovery assessed by the Fugl-Meyer Assessment (FMA) upper/lower extremity subscale scores measured immediately after the 2-week intervention period. Secondary clinical endpoints included functional independence measured by the Modified Barthel Index (MBI) and motor recovery staging assessed by Brunnstrom stages (upper and lower limbs). Neuroimaging measures derived from resting-state fNIRS were considered exploratory outcomes. All clinical assessments were performed by an experienced rehabilitation physician who was blinded to group allocation and did not participate in intervention delivery.

#### 2.4.1. Primary endpoint: Fugl-Meyer Assessment (FMA).

Motor recovery was evaluated using the upper- and lower-extremity subscales of the Fugl-Meyer Assessment (FMA). These standardized and widely validated instruments assess motor impairment in individuals with stroke [21,22].

#### 2.4.2. Secondary clinical endpoints: Modified Barthel Index (MBI) and Brunnstrom Stages.

Secondary clinical endpoints included functional independence assessed by the Modified Barthel Index (MBI) and motor recovery staging evaluated by Brunnstrom stages (upper and lower limbs). The MBI assesses functional independence in activities of daily living, while Brunnstrom stages categorize motor recovery progression. These assessments were conducted at baseline and immediately after the 2-week intervention period.

#### 2.4.3. Exploratory neuroimaging outcomes (fNIRS).

Resting-state cortical activity and FC were assessed as secondary, exploratory outcomes using a continuous-wave fNIRS system (NirSmart; Danyang HuiChuang Medical Equipment Co., Ltd.) operating at dual wavelengths (730 and 850 nm) with a sampling rate of 10 Hz. The optode montage comprised 39 measurement channels covering prefrontal, sensorimotor, and motor cortical regions, with source-detector separations ranging from 2.7 to 3.3 cm. During data acquisition, participants were instructed to sit quietly with eyes open and fixate on a blank wall for 7 minutes [23].

Raw optical density signals were corrected for motion artifacts and converted to changes in oxygenated HbO concentration using the modified Beer-Lambert law. Signals were detrended to remove baseline drifts, and residual systemic and motion-related noise was attenuated using short-separation regression when available. Subsequently, band-pass filtering (0.01–0.08 Hz) was applied to isolate low-frequency spontaneous fluctuations relevant to resting-state activity. All preprocessing procedures were performed using NIRSPARK and NIRS-KIT software packages.

Fractional amplitude of low-frequency fluctuations (fALFF) was calculated from preprocessed oxygenated HbO signals within the 0.01–0.08 Hz frequency band using fast Fourier transform. fALFF values were Z-standardized within participants and averaged across channels corresponding to predefined regions of interest, including the ipsilesional primary motor cortex, sensorimotor cortex, and primary somatosensory cortex [24]. FC was assessed by computing Pearson correlation coefficients between region-of-interest time series, generating ROI-to-ROI connectivity matrices that were subsequently Fisher Z-transformed prior to group-level statistical analysis [25].

## 3. Statistical analysis

**Sample size considerations:** This head-to-head comparison represents a secondary analysis of a completed three-arm randomized controlled trial. The original trial was powered for the primary comparison of neuromodulation interventions versus conventional rehabilitation, not specifically for direct LIFU versus rTMS comparisons. Post-hoc power calculation revealed that the available sample size (n = 25 per group) achieves approximately 78% power to detect a medium effect size (Cohen’s d = 0.5) at α = 0.05 (two-tailed), which approaches but does not reach the conventional 80% standard. Consequently, between-group findings should be interpreted as exploratory, and null results do not exclude the possibility of clinically meaningful differences.

Statistical analyses were performed using IBM SPSS Statistics (version 31.0; IBM Corp., Armonk, NY, USA). Normality was assessed using the Shapiro–Wilk test. Continuous variables are presented as mean ± SD or median (IQR) and were analyzed using paired or independent t-tests, or Wilcoxon signed-rank and Mann–Whitney U tests, as appropriate.

For clinical outcomes, within-group changes from baseline to post-intervention were assessed first. Between-group comparisons were primarily based on post-intervention scores. Change-from-baseline FMA scores (ΔFMA) were analyzed as complementary measures of improvement magnitude. Brunnstrom stages, treated as ordinal outcomes, were analyzed using nonparametric tests.

Resting-state fNIRS metrics were analyzed as secondary, exploratory outcomes. For functional connectivity analyses, p-values were adjusted for multiple comparisons using the Benjamini–Hochberg false discovery rate procedure. For the primary clinical endpoints (FMA upper extremity and FMA lower extremity scores), in addition to between-group comparisons based on post-intervention scores, analysis of covariance (ANCOVA) was performed as a supplementary analysis to adjust for the potential influence of baseline values on post-intervention outcomes. The ANCOVA model included post-intervention scores as the dependent variable, treatment group as a fixed factor, and baseline scores of the respective measure as a covariate. Prior to analysis, the assumption of homogeneity of regression slopes was examined; results indicated no significant interaction between group and baseline scores (all *p* > 0.05), satisfying the conditions for ANCOVA application. This analysis was conducted to assess the potential impact of baseline differences on between-group comparisons and to serve as a sensitivity verification of the primary analysis results. All statistical tests were two-tailed, with p < 0.05 considered statistically significant.

## 4. Results

### 4.1. Participant flow and baseline characteristics

The original three-arm trial randomized 75 participants (25 to each group: standard rehabilitation alone, LIFU, or rTMS). For the present secondary head-to-head comparison, participants allocated to the standard rehabilitation-only group (n = 25) were excluded, and analyses were conducted on the remaining 50 participants randomized to the two neuromodulation arms (LIFU: n = 25; rTMS: n = 25). Of the 50 randomized participants, 43 (86.0%) completed the 2-week intervention and all scheduled assessments and were included in the per-protocol (completer) analysis (LIFU: 21/25; rTMS: 22/25). Seven participants (14.0%) discontinued the study prematurely because of hospital transfer (n = 4), financial reasons (n = 2), or personal decision (n = 1); none of these withdrawals were related to the intervention or to adverse events.

The primary analyses were conducted in the intention-to-treat (ITT) population (n = 50), with missing post-intervention data for participants who discontinued handled using the last observation carried forward approach. Per-protocol analyses of the 43 participants who completed the intervention were performed as sensitivity analyses. Overall, the per-protocol results were consistent with the ITT analyses in terms of the direction of change and the main between-group conclusions for post-intervention clinical outcomes, although statistical significance for some analyses differed between populations (Supplementary Tables 1–3 in S1 File).

**Fig 1:**
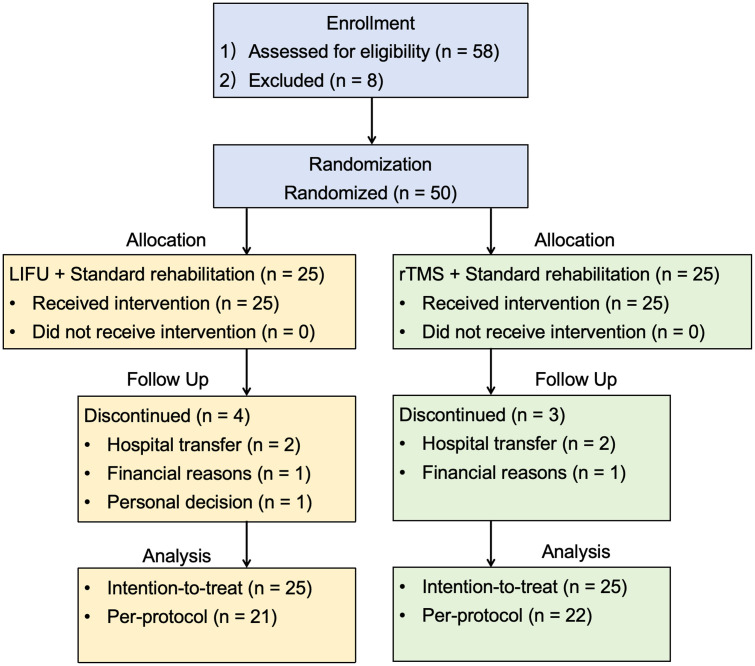
CONSORT flow diagram of participant progress through the present secondary analysis.

Baseline demographic and clinical characteristics were comparable between the two intervention groups in both the ITT population (Tables 1 and 2) and the completer sample (Supplementary Tables 1 and 2 in S1 File). Participant flow through the study is summarized in the CONSORT diagram shown in Fig 1.

**Table 1:** Baseline demographic characteristics (ITT population, n = 50).

	Age, years	Sex		Disease Duration, weeks	Hemiplegia Side		Stroke Type	
		Male	Female		Left	Right	Infarction	Hemorrhage
**LIFU Group (n = 25)**	64.32 ± 9.37	19	6	3.14 ± 4.18	13	12	17	8
**rTMS Group (n = 25)**	64.20 ± 9.41	17	8	3.26 ± 4.15	14	11	16	9
**Statistical Value** **vs. between groups**	t = 0.05, *p* = 0.96; Cohen’s d = 0.013, 95% CI [−0.54, 0.57]	χ² = 0.40, *p* = 0.53		t = −0.10, *p* = 0.92 Cohen’s d = −0.03, 95% CI [−0.58, 0.52]	χ² = 0.08, *p* = 0.78		χ² = 0.09, *p* = 0.77	

**Note:** Data are mean ± SD, median (IQR), or n (%). p-values were calculated by independent-samples t-test, Mann-Whitney U test, χ² test, or Fisher’s exact test as appropriate. No significant differences were observed between groups (p > 0.05 for all comparisons).

**Table 2:** Clinical outcome characteristics (ITT population, n = 50).

	FMA		MBI (Points/100)	Brunnstrom Stage (Stages/6)	
	Upper Limb (Points/66)	Lower Limb (Points/34)		Upper Limb	Lower Limb
**LIFU Group (n = 25)**					
**Pre-Training**	32.00 ± 21.75	22.68 ± 8.33	51.00 ± 21.26	4 (3-5)	4 (3-5)
**Post-Training**	40.60 ± 21.26*	26.60 ± 7.50*	73.20 ± 18.65*	4.5(4-5)*	5 (4-5)*
**Statistical Value** **vs. Pre- and Post-**	t = −5.61, *p <* 0.001; Cohen’s d = −1.12, 95% CI [−1.62, −0.61]	t = −5.40, *p <* 0.001; Cohen’s d = −1.08, 95% CI [−1.57, −0.58]	t = −9.32, *p <* 0.001; Cohen’s d = −1.86, 95% CI [−2.51, − 1.20]	*Z* = −3.70, *p* *<* 0.001	*Z* = −3.07, *p* = 0.002
**rTMS Group (n = 25)**					
**Pre-Training**	32.20 ± 22.10	23.96 ± 6.88	50.28 ± 24.46	4 (2–5)	4 (3–5)
**Post-Training**	35.28 ± 22.44*	25.24 ± 6.93*	72.00 ± 19.53*	4 (3–5)*	5 (4–5)*
**Statistical Value** **vs. Pre- and Post-**	t = −4.29, *p <* 0.001; Cohen’s d = −0.86, 95% CI [−1.31, −0.39]	t = −4.31, *p <* 0.001; Cohen’s d =−0.86, 95% CI [−1.32, −0.39]	t = −6.12, *p <* 0.001; Cohen’s d = −1.22, 95% CI [−1.74, −0.69]	*Z* = −2.57, *p* = 0.01	*Z* = −2.53, *p* = 0.011
**Statistical Value** **vs. Pre- groups**	t = −0.03, *p* = 0.97; Cohen’s d = −0.01, 95% CI [−0.56, 0.55]	t = −0.59, *p* = 0.56; Cohen’s d = −0.17, 95% CI [−0.72, 0.39]	t = 0.11, *p* = 0.91; Cohen’s d = 0.03, 95% CI [−0.52, 0.59]	*Z* = −0.40, *p* = 0.97	*Z* = −0.58, *p* = 0.58
**Statistical Value** **vs. Post- groups**	t = 0.86, *p* = 0.39; Cohen’s d = 0.24, 95% CI [−0.31, 0.80]	t = 0.67, *p* = 0.51; Cohen’s d = 0.19, 95% CI [−0.37, 0.74]	t = 0.22, *p* = 0.83; Cohen’s d = 0.06, 95% CI [−0.49, 0.62]	*Z* = −0.97, *p* = 0.33	*Z* = −0.18, *p* = 0.85

**Note:** Data are presented as mean ± SD, median (IQR), or n (%). *p < 0.05 indicates a statistically significant pre- to post-intervention difference within groups.

Fifty participants (selected from the original three-arm trial of 75 participants) has been randomized to receive standard rehabilitation combined with either low-intensity focused ultrasound (LIFU, n = 25) or repetitive transcranial magnetic stimulation (rTMS, n = 25). The original trial included a third group receiving standard rehabilitation alone (n = 25), which was excluded from the present secondary analysis. During the 2-week intervention period, seven participants discontinued the study (hospital transfer, n = 4; financial reasons, n = 2; personal decision, n = 1), none due to intervention-related adverse events. A total of 43 participants completed the intervention and post-treatment assessments and were included in the per-protocol analysis (LIFU, n = 21; rTMS, n = 22). The primary analyses were conducted in the ITT population (n = 50), with missing post-intervention data handled using last observation carried forward imputation.

### 4.2. Clinical outcomes

Baseline motor function did not differ significantly between the LIFU and rTMS groups, as indicated by comparable FMA scores for the upper and lower extremities, MBI scores, and Brunnstrom stages for both limbs (Table 2).

Following the 2-week intervention, both groups demonstrated significant within-group improvements in motor impairment and functional independence. Specifically, FMA scores for both the upper and lower extremities and MBI scores improved significantly in the LIFU group (all p < 0.001) and in the rTMS group (p = 0.001 or p < 0.001). Improvements in Brunnstrom stages were observed across limbs or groups. In the LIFU group, significant improvements were observed in both the upper- and lower-limb Brunnstrom stage (p < 0.001 and p = 0.002, respectively). In the rTMS group, both upper- and lower-limb Brunnstrom stages showed statistically significant changes over the intervention period (p = 0.01 and p = 0.011, respectively).

Despite these within-group gains, between-group comparisons based on post-intervention scores revealed no statistically significant differences between the LIFU and rTMS groups for FMA (upper or lower extremities), MBI, or Brunnstrom stages (all p > 0.05; Table 2). Effect size estimates (Cohen’s d) for FMA and MBI and nonparametric test statistics for Brunnstrom stages are provided in Table 2.

To further control for the potential influence of baseline values on post-intervention outcomes, analysis of covariance (ANCOVA) was performed as a supplementary analysis for FMA upper and lower extremity scores. Results showed that after adjusting for baseline upper-extremity FMA scores, the between-group difference in post-intervention upper-extremity FMA scores was statistically significant (*F* = 9.901, *p* = 0.003); however, after adjusting for baseline lower-extremity FMA scores, the between-group difference in post-intervention lower-extremity FMA scores did not reach statistical significance (*F* = 3.299, *p* = 0.076) (Supplementary Table 1 in S2 File). These findings were not entirely consistent with the primary analyses based on post-intervention scores alone (upper extremity: *p* = 0.39; lower extremity: *p* = 0.51), suggesting that baseline upper-extremity FMA scores may have influenced the comparison of post-intervention upper-extremity outcomes.

To further characterize the magnitude of motor improvement over time, change-from-baseline scores for FMA (ΔFMA) were analyzed as a complementary outcome. In the ITT analysis, the LIFU group exhibited greater median improvements in both upper- and lower-extremity FMA scores compared with the rTMS group (Table 3). Similar directional findings were observed in the completer (per-protocol) sample (Supplementary Table 3 in S1 File). These change-score analyses did not alter the primary between-group conclusions based on post-intervention scores but provide additional context regarding differences in the extent of motor recovery during the intervention period.

**Table 3:** Clinical outcomes (FMA improvement) characteristics (ITT population, n = 50).

	Δ FMA	
	Upper Limb	Lower Limb
**LIFU Group (n = 25)**	7 (3–10.5)	3 (1–4.5)
**rTMS Group (n = 25)**	2 (1–3)*	1 (0–1.5)*
**Statistical Value vs. between groups**	*Z* = –3.23, *p* = 0.001	*Z* = –3.84, *p <* 0.001

**Note:** Data are presented as median (IQR). *p < 0.05 indicates a statistically significant between-group difference.

### 4.3. Secondary neuroimaging outcomes

Resting-state fNIRS data were obtained at baseline and after the 2-week intervention period in all participants who completed the study protocol. Neuroimaging analyses were therefore conducted using complete-case data without imputation (LIFU, n = 21; rTMS, n = 22).

**Fig 2:**
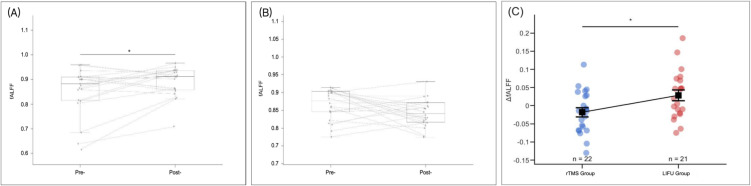
Resting-state fALFF and ΔfALFF following the 2-week intervention. **(A)** Prefrontal fALFF increased from baseline to post-intervention in the LIFU group (paired t-test, p = 0.002, Cohen’s d = 0.54). **(B)** No statistically significant change in prefrontal fALFF was observed in the rTMS group (paired t-test, p = 0.067, Cohen’s d = −0.41). **(C)** The ΔfALFF in motor-related regions differed between groups, with greater values observed in the LIFU group compared with the rTMS group (independent-samples t-test, t = 2.44, p = 0.019, Cohen’s d = 0.74). Each circle represents an individual participant. Data are presented as mean ± SD. *p < 0.05 indicates a statistically significant within-group or between-group comparison.

fALFF was analyzed as an index of spontaneous cortical activity. In the LIFU group, prefrontal fALFF increased significantly after the intervention compared with baseline (p = 0.002, Cohen’s d = 0.54; Fig 2A). In contrast, no statistically significant change in prefrontal fALFF was observed in the rTMS group (p = 0.067, Cohen’s d = −0.41; Fig 2B). The between-group comparison showed a greater change in fALFF within motor-related cortical regions in the LIFU group than in the rTMS group (0.028 ± 0.067 vs. −0.018 ± 0.059; t = 2.44, p = 0.019, Cohen’s d = 0.74; Fig 2C).

**Fig 3:**
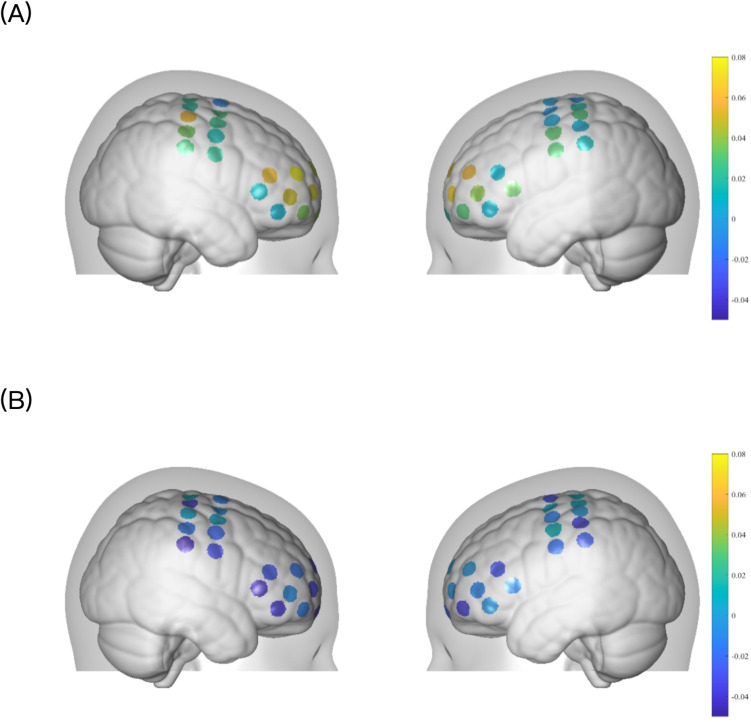
3D channel-wise topographic maps of group-averaged ΔfALFF following the 2-week intervention. **(A)** LIFU group and **(B)** rTMS group. Color scale represents group-averaged ΔfALFF. Maps are rendered without spatial interpolation on a standardized head model.

Channel-wise group-averaged ΔfALFF distributions were visualized using non-interpolated three-dimensional topographic mapping to illustrate spatial patterns of cortical activity changes (Fig 3). These maps are presented for descriptive purposes only; no channel-wise comparisons remained statistically significant after correction for multiple comparisons.

**Fig 4:**
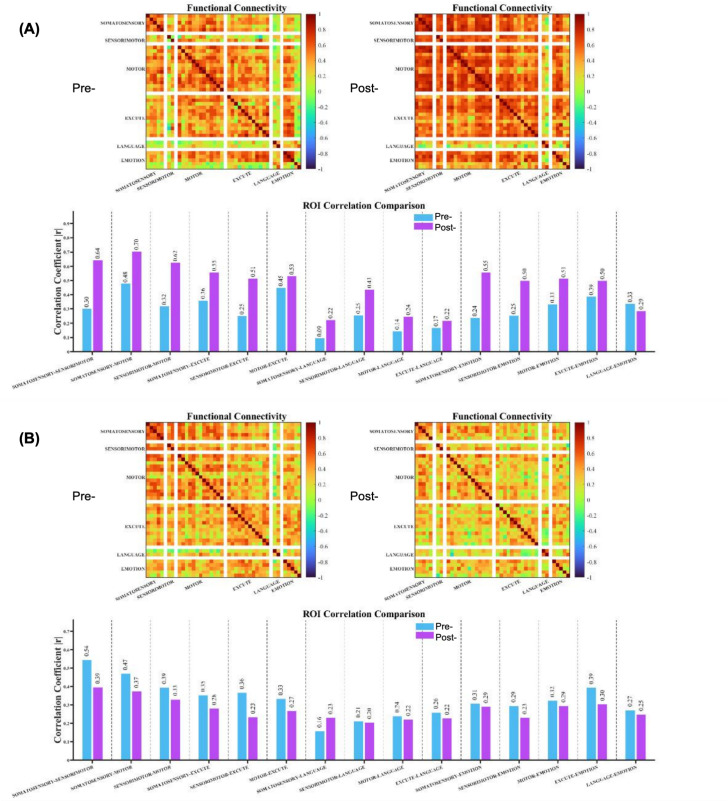
Resting-state FC matrices following the 2-week intervention. **(A)** LIFU group and **(B)** rTMS group. Group-averaged ROI-to-ROI Pearson correlation matrices (Fisher z-transformed) are shown before (left) and after (right) the intervention. ROI were ordered according to six predefined ipsilesional networks: somatosensory, sensorimotor, motor, executive, language, and emotion. Color scales represent correlation strength, with warmer colors indicating higher positive correlations and cooler colors indicating lower or negative correlations. Lower panels depict absolute correlation coefficients (|r|) for all ROI pairs before and after the intervention. No connectivity changes remained statistically significant after false discovery rate correction (all adjusted p > 0.05).

Resting-state FC analyses revealed distinct patterns of change between groups. In the LIFU group, ROI-to-ROI correlation matrices suggested increased inter-regional coupling in motor, sensorimotor, and executive networks following the intervention, whereas the rTMS group exhibited an overall tendency toward reduced connectivity (Fig 4). However, none of the within-group or between-group connectivity changes reached statistical significance after false discovery rate correction (all adjusted p > 0.05). No treatment-related adverse events were observed in either group during the study period [26].

### 4.4. Safety outcomes

No stimulation-related adverse events were observed in either intervention group throughout the study period. All participants tolerated the complete course of LIFU or rTMS without serious adverse events, neurological complications, or treatment interruptions.

## 5. Discussion

Increased prefrontal fALFF following LIFU was observed in the present study; however, in the absence of task-based fNIRS data demonstrating functional engagement, correlational analyses linking fALFF changes to clinical outcomes, or longitudinal data establishing the persistence of these changes, this finding should be interpreted cautiously. We speculate that elevated prefrontal fALFF may reflect potential alterations in local spontaneous neuronal activity within executive and associative cortical regions involved in motor planning, attention, and error monitoring—functions that have been proposed as compensatory mechanisms when primary motor pathways are compromised [27,28]. Nevertheless, given that this study was limited to resting-state measurements without task-based functional assessment, behavioral correlation, or follow-up evaluation, the observed fALFF changes cannot be construed as evidence of enhanced functional engagement, nor can they support causal inferences regarding neural mechanisms underlying motor recovery. The relationship between prefrontal fALFF elevation and compensatory cortical recruitment remains hypothetical and requires validation through task-based neuroimaging, outcome-associated longitudinal designs, and mechanistic studies in future research. Clinically, both intervention groups demonstrated significant within-group improvements in motor impairment and functional independence, as reflected by gains in FMA scores for the upper and lower extremities and improvements in MBI scores following the 2-week intervention period. Changes in Brunnstrom stages were consistent, with significant improvements observed in both upper- and lower-limb Brunnstrom stages in the LIFU and rTMS groups. These findings are consistent with prior studies showing that adjunctive neuromodulation during the subacute phase of stroke can support short-term improvements in impairment- and disability-related outcomes when combined with conventional rehabilitation [21,22].

Despite these within-group gains, no statistically significant between-group differences were observed in post-intervention clinical scores. This result aligns with previous neuromodulation studies conducted in the subacute stage of stroke recovery, during which spontaneous biological recovery and intensive standard rehabilitation often contribute substantially to early functional gains. Under such conditions, detecting between-group differences based solely on post-intervention behavioral measures over relatively short intervention periods may be challenging [29].

In addition to the primary analysis, this study performed analysis of covariance (ANCOVA) to adjust for the potential influence of baseline values on post-intervention outcomes. Results showed that after adjusting for baseline upper-extremity FMA scores, the between-group difference in post-intervention upper-extremity FMA scores reached statistical significance (*p* = 0.003), whereas the primary analysis based on post-intervention scores alone did not reveal significant differences (*p* = 0.39). This discrepancy may be attributable to the following factors: although randomization achieved overall balance in baseline characteristics between groups, baseline differences in upper-extremity FMA scores—despite not reaching statistical significance (Table 2)—may have nevertheless influenced post-intervention comparisons. By controlling for baseline variance, ANCOVA increased statistical power and revealed potential between-group differences that were not apparent in the unadjusted analysis. This finding suggests that baseline-adjusted analytical approaches may facilitate more sensitive detection of intervention effects in future studies. Nonetheless, the primary conclusions of this study remain based on the pre-specified primary analytical method (post-intervention score comparison), and the ANCOVA results for lower-extremity FMA scores were consistent with the primary analysis (*p* = 0.076). Therefore, the overall robustness of the study conclusions is not fundamentally affected.

In contrast, analyses of change-from-baseline FMA scores revealed greater motor improvements in the LIFU group compared with the rTMS group. However, this apparent discrepancy—significant ΔFMA differences yet non-significant post-intervention score comparisons—should be interpreted cautiously. As this study represents a secondary head-to-head comparison that was not independently powered to detect between-group differences between LIFU and rTMS, the observed pattern may reflect limited statistical power rather than true treatment equivalence. Although baseline FMA scores were comparable between groups, the modest sample size (n = 25 per group) provides approximately 78% power to detect a medium effect size (Cohen’s d = 0.5), approaching but not reaching the conventional 80% threshold. Consequently, the lack of significant between-group differences in post-intervention scores may indicate insufficient sensitivity to detect subtle endpoint differences, rather than confirming comparable efficacy. Importantly, these differences in ΔFMA did not translate into significant between-group differences in post-intervention scores and should therefore not be interpreted as evidence of superior short-term clinical efficacy. Rather, ΔFMA may reflect differences in recovery trajectories or rates of motor improvement during the intervention period. In the subacute phase of stroke, baseline variability, spontaneous recovery, and potential ceiling effects may limit the sensitivity of post-intervention comparisons, whereas change-score analyses can provide complementary information regarding recovery dynamics without supplanting post-treatment outcomes as the primary basis for group comparison. All between-group findings should be considered exploratory and require confirmation in larger, adequately powered prospective head-to-head trials.

Although short-term clinical outcomes were comparable between groups, resting-state fNIRS analyses revealed modality-specific patterns of cortical activity. LIFU was associated with significant increases in fALFF and greater changes in motor-related cortical regions, whereas rTMS did not induce statistically significant changes in these measures. Functional connectivity analyses further suggested differing directional trends between groups; however, none of these connectivity changes remained significant after correction for multiple comparisons. These neuroimaging findings, while exploratory, suggest that LIFU and rTMS may differentially modulate cortical activity during early recovery.

Such differences in cortical activity patterns do not necessarily imply superior clinical efficacy but may reflect distinct neural strategies or compensatory mechanisms engaged during motor recovery. Increased prefrontal fALFF following LIFU may indicate enhanced engagement of executive and associative cortical regions involved in motor planning, attention, and error monitoring, which have been proposed as compensatory mechanisms when primary motor pathways are compromised [29,30]. Conversely, the absence of significant fALFF changes following rTMS may reflect a more focal modulation of primary motor regions with less reliance on higher-order cortical recruitment [31]. Given the exploratory nature of these analyses and the lack of statistically significant connectivity changes after multiple-comparison correction, these interpretations should be considered hypothesis-generating rather than confirmatory.

Taken together, the present findings suggest that LIFU and rTMS yield comparable short-term functional outcomes in subacute stroke while potentially engaging cortical networks in different ways. Differences observed in neuroimaging measures and recovery trajectories may reflect early neural adaptations that precede stable behavioral differentiation. Whether such early neural differences translate into long-term functional advantages requires confirmation in larger studies with extended follow-up and multimodal neuroimaging approaches [32,33].

This study has several limitations. First, the inclusion criterion of stroke onset within 6 months encompasses the acute, early subacute, and late subacute/early chronic phases, which could introduce heterogeneity in pathophysiological states and recovery trajectories. However, baseline characteristics—including time since stroke onset, age, sex, and initial FMA scores—were well balanced between groups (all *P* > 0.05), suggesting that potential confounding from disease stage variability was minimized in our between-group comparisons. Nonetheless, due to the fixed sample size of this completed randomized controlled trial, we were unable to conduct subgroup analyses stratified by specific stroke phases (e.g., < 1 month vs. 1–3 months vs. 3–6 months). Future studies with larger cohorts or more narrowly defined inclusion windows (e.g., 1–3 months post-stroke) are warranted to validate whether our findings generalize across distinct recovery phases and to elucidate potential phase-specific treatment effects. Second, the modest sample size and short intervention duration may have limited statistical power, particularly for detecting subtle or distributed changes in functional connectivity in the context of post-stroke heterogeneity. Third, as a secondary head-to-head analysis, lesion-specific or subtype-stratified effects were not examined. In addition, fNIRS is limited to superficial cortical regions and remains susceptible to extracerebral physiological noise, restricting inferences regarding subcortical mechanisms. Future studies with larger cohorts, longer follow-up periods, and integrated neuroimaging modalities are warranted to validate and extend these findings. Fourth, the observed pattern of significant between-group differences in change-from-baseline FMA scores (ΔFMA) alongside non-significant differences in post-intervention endpoint scores warrants careful interpretation. This secondary head-to-head comparison was not prospectively powered independently to detect between-group differences between LIFU and rTMS. Post-hoc power analysis indicated approximately 78% power to detect a medium effect size (Cohen’s d = 0.5), approaching but not reaching the conventional 80% threshold. This marginal statistical power may have been insufficient to detect subtle differences in post-intervention endpoint scores, even when change-from-baseline analyses suggested differential recovery trajectories. Therefore, between-group comparisons should be considered exploratory, and the lack of significant differences in endpoint scores should not be interpreted as confirming comparable efficacy between the two interventions.

## 6. Conclusion

When combined with standard rehabilitation, LIFU and rTMS were associated with comparable short-term motor outcomes in individuals with subacute stroke. Exploratory fNIRS analyses indicated modality-specific patterns of cortical activity, whereas functional connectivity changes did not reach statistical significance. These findings suggest potential differences in neural modulation without evidence of differential short-term clinical efficacy. Larger, longitudinal studies are required to clarify the underlying mechanisms and determine their long-term clinical implications.

## Supporting information

S1 File Supplementary Tables S1–S3 present per-protocol (completer) analyses of the 43 participants who completed all intervention sessions and assessments. These sensitivity analyses were performed to verify the robustness of the primary intention-to-treat findings. Results were consistent with the ITT analysis in terms of the direction of change and the main between-group conclusions for post-intervention clinical outcomes.
(DOCX)

S2 File This table presents the results of supplementary analysis of covariance (ANCOVA) performed to adjust for the potential influence of baseline FMA scores on post-intervention outcomes. The ANCOVA model included post-intervention FMA scores as the dependent variable, treatment group as a fixed factor, and baseline FMA scores as a covariate. After adjusting for baseline upper-extremity FMA scores, the between-group difference in post-intervention upper-extremity FMA scores was statistically significant (F = 9.901, *p* = 0.003). However, after adjusting for baseline lower-extremity FMA scores, the between-group difference in post-intervention lower-extremity FMA scores did not reach statistical significance (F = 3.299, *p* = 0.076). These findings suggest that baseline upper-extremity motor function may have influenced the comparison of post-intervention outcomes between groups.
(XLSX)

## Abbreviations

fALFF: fractional amplitude of low-frequency fluctuations
FC: functional connectivity
fNIRS: functional near-infrared spectroscopy
FMA: Fugl-Meyer assessment
ISPPA: spatial-peak pulse-average intensity
ITT: the intention-to-treat
LIFU: low-intensity focused ultrasound
MBI: Modified Barthel Index
ROI: region of interest
rTMS: repetitive transcranial magnetic stimulation
