# Supplementary material for: A secondary head-to-head comparison of low-intensity focused ultrasound and repetitive transcranial magnetic stimulation for motor recovery after stroke
Source: PLoS One. 2026 Apr 24;21(4):e0348030. doi: 10.1371/journal.pone.0348030 (PMC13108770; doi:10.1371/journal.pone.0348030)
Supplement: S1 File — These sensitivity analyses were performed to verify the robustness of the primary intention-to-treat findings. Results were consistent with the ITT analysis in terms of the direction of change and the main between-group conclusions for post-intervention clinical outcomes. (DOCX) [file pone.0348030.s001.docx]

**Table S1. Baseline demographic in participants who completed the study (n=43)**

|  | **Age, years** | **Sex** | | | **Disease Duration, weeks** | **Hemiplegia Side** | | **Stroke Type** | |
| --- | --- | --- | --- | --- | --- | --- | --- | --- | --- |
|  |  | **Male** | **Female** | |  | **Left** | **Right** | **Infarction** | **Hemorrhage** |
| **LIFU Group (n = 21)** | 64.86 ± 9.08 | 15 | 6 | 3.36 ± 4.52 | | 12 | 9 | 16 | 5 |
| **rTMS Group (n = 22)** | 64.59 ± 9.93 | 14 | 8 | 3.43 ± 4.39 | | 13 | 9 | 15 | 7 |
| **Statistical Value**  **vs. between groups** | t = 0.09, *p* = 0.93;  Cohen’ d = 0.03,  95% CI [-0.57, 0.63] | χ² = 0.30, *p* = 0.59 | | | t = -0.05, *p* = 0.96  Cohen’ d = -0.02,  95% CI [-0.61, 0.58] | χ² = 0.02, *p* = 0.90 | | χ² = 0.34, *p* = 0.56 | |

**Note:** Data are mean ± SD, median (IQR), or n (%). *p*-values were calculated by independent-samples t-test, Mann-Whitney U test, χ² test, or Fisher’s exact test as appropriate. No significant differences were observed between groups (*p* > 0.05 for all comparisons).

**Table S2. Clinical outcomes in participants who completed the study (n=43)**

|  | **FMA** | | **MBI**  **（Points /100）** | **Brunnstrom Stage（Stages /6）** | |
| --- | --- | --- | --- | --- | --- |
|  | **Upper Limb（Points /66）** | **Lower Limb（Points /34）** |  | **Upper Limb** | **Lower Limb** |
| **LIFU Group (n = 21)** | | | | | |
| **Pre-Training** | 32.62±22.77 | 22.81±8.47 | 51.67±21.06 | 4 (3-5) | 4 (3-5) |
| **Post-Training** | 41.29±22.26＊ | 26.95±7.45＊ | 74.05±18.82＊ | 5(4-6)＊ | 5 (4-5)＊ |
| **Statistical Value**  **vs. Pre- and Post-** | t = -4.78, *p <* 0.001;  Cohen’ d = -1.04,  95% CI [-1.57, -0.50] | t =- 4.87, *p <* 0.001;  Cohen’ d = -1.06,  95% CI [-1.59, -0.52] | t = -8.01, *p <* 0.001;  Cohen’ d =- 1.75,  95% CI [-2.43,- 1.05] | *Z* = -3.17, *p* = 0.002 | *Z* = -3.07, *p* = 0.002 |
| **rTMS Group (n = 22)** | | | | | |
| **Pre-Training** | 33.55±22.63 | 24.77±6.41 | 52.82±23.86 | 4 (2.75-5) | 4 (4-5) |
| **Post-Training** | 36.64±22.95＊ | 26.14±6.42＊ | 75.23±16.51＊ | 4.5 (3-5)＊ | 5 (4-5.25)＊ |
| **Statistical Value**  **vs. Pre- and Post-** | t = -3.79, *p =* 0.001;  Cohen’ d = -0.81,  95% CI [-1.28, -0.32] | t = -4.10, *p <* 0.001;  Cohen’ d = -0.87,  95% CI [-1.36, -0.37] | t = -5.58, *p <* 0.001;  Cohen’ d = -1.19,  95% CI [-1.73, -0.63] | *Z* = -2.40, *p* = 0.016 | *Z* = -2.53, *p* = 0.011 |
| **Statistical Value**  **vs. Pre- groups** | t = -0.13, *p* = 0.89;  Cohen’ d =- 0.04,  95% CI [-0.64, 0.56] | t = -0.86, *p* = 0.40;  Cohen’ d = -0.26,  95% CI [-0.86, 0.34] | t = -0.17, *p* = 0.87;  Cohen’ d = -0.05,  95% CI [-0.65, -0.55] | *Z* = -0.06, *p* = 0.95 | *Z* = -1.13, *p* = 0.26 |
| **Statistical Value**  **vs. Post- groups** | t = 0.67, *p* = 0.50;  Cohen’ d = 0.21,  95% CI [-0.40, 0.80] | t = 0.39, *p* = 0.70;  Cohen’ d = 0.12,  95% CI [-0.48, 0.72] | t = -0.22, *p* = 0.83;  Cohen’ d = -0.07,  95% CI [-0.66, 0.53] | *Z* = -0.82, *p* = 0.41 | *Z* = -0.19, *p* = 0.85 |

**Note:** Data are presented as mean ± SD or n (%). ＊*p* < 0.05 indicates a statistically significant pre- to post-intervention difference between groups.

**Table S3. Clinical outcomes (FMA improvement) characteristics (n=43)**

|  | Δ **FMA** | |
| --- | --- | --- |
|  | **Upper Limb** | **Lower Limb** |
| **LIFU Group (n = 21)** | 7 (3-11) | 3 (1-5) |
| **rTMS Group (n = 22)** | 2 (1-3)＊ | 1 (0-2)＊ |
| **Statistical Value**  **vs. between groups** | *Z* = -2.80, *p* = 0.01 | *Z* = -3.38, *p <* 0.001 |

**Note:** Data are presented as median (IQR). ＊*p* < 0.05 indicates a statistically significant between-group differences.
